# Mechanisms of protein-folding diseases at a glance

**DOI:** 10.1242/dmm.013474

**Published:** 2014-01

**Authors:** Julie S. Valastyan, Susan Lindquist

**Affiliations:** 1 Whitehead Institute for Biomedical Research, Cambridge, MA 02142, USA.; 2 Department of Biology, Massachusetts Institute of Technology, Cambridge, MA 02139, USA.; 3 Howard Hughes Medical Institute, Massachusetts Institute of Technology, Cambridge, MA 02139, USA.

**Keywords:** Disease, Misfolding, Protein, Yeast

## Abstract

For a protein to function appropriately, it must first achieve its proper conformation and location within the crowded environment inside the cell. Multiple chaperone systems are required to fold proteins correctly. In addition, degradation pathways participate by destroying improperly folded proteins. The intricacy of this multisystem process provides many opportunities for error. Furthermore, mutations cause misfolded, nonfunctional forms of proteins to accumulate. As a result, many pathological conditions are fundamentally rooted in the protein-folding problem that all cells must solve to maintain their function and integrity. Here, to illustrate the breadth of this phenomenon, we describe five examples of protein-misfolding events that can lead to disease: improper degradation, mislocalization, dominant-negative mutations, structural alterations that establish novel toxic functions, and amyloid accumulation. In each case, we will highlight current therapeutic options for battling such diseases.

## Introduction

Proteins are the molecular machines that control our most vital cellular functions. To fulfill its role, a protein must first fold into its correct three-dimensional structure, assuming complicated tertiary and sometimes quaternary conformations. Although many aspects of folding are intrinsic to the biophysical properties of the protein itself, the process is quite complex and susceptible to errors (Dill and MacCallum, 2012). Proteins consist of an elaborate arrangement of interior folds that collapse into a final thermodynamically stable structure and, for many proteins, only a modest free-energy gain (generally only −3 to −7 kcal/mol) (Lindquist and Kelly, 2011) is associated with correct folding of a protein compared with its innumerable potential misfolded states. Thereby, the latter can sometimes be favored (see poster panel 1). Furthermore, many misfolded proteins involved in disease contain one or more mutations that destabilize the correct fold and/or stabilize a misfolded state. *In vivo*, protein folding is made even more difficult by the crowded environment of the cell, where proteins must assume their correct conformation while being constantly bombarded by high-energy collisions with neighboring proteins (Ellis and Minton, 2006). These complications make it no surprise that many proteins do not achieve their correct conformations, or stably assume the wrong ones. Both of these scenarios can result in disease. Furthermore, in eukaryotic cells, protein folding must occur in several distinct compartments: in addition to the small, specialized organelles such as mitochondria and peroxisomes, there are the massive compartments of the endoplasmic reticulum (ER) for membrane and secreted proteins, as well as the cytosol and nucleus. The very different chemical nature of these compartments results in different protein-folding problems that each cell must prevent and address.

Cells have multiple methods for combating these problems. First, chaperones are expressed constitutively and further induced in response to the accumulation of unfolded proteins. In the ER, this response is known as the unfolded protein response (UPR); in the nuclear and cytosolic compartment it is known as the heat-shock response (HSR). (Other organelles have additional responses, which remain poorly characterized.) Initially characterized as emergency responses to sudden stresses, it is now apparent that these responses are constantly responding to small perturbations in protein homeostasis and play vital roles in helping proteins become folded in the first place or in aiding misfolded proteins to regain their correct conformation (reviewed in Hartl et al., 2011). Second, when it becomes clear that a misfolded protein cannot be properly refolded, systems, such as the proteasome, autophagy and ER-associated degradation (ERAD), are deployed to degrade these misfolded proteins (reviewed in Nedelsky et al., 2008; Smith et al., 2011; Varshavsky, 2012). Dysfunction of any of these pathways can, unsurprisingly, lead to protein-misfolding disease. This is by no means the sole mechanism, however.

The first known protein-misfolding disease, indeed the first inherited human disease to have a known molecular mechanism, was sickle cell anemia. In this disorder, a single point mutation changes a glutamic acid in the β-globulin chain of hemoglobin into a valine (Ingram, 1957; Hunt and Ingram, 1959). In the deoxygenated environment of tissue capillary beds (Gibson and Ellory, 2002), the protein changes conformation, exposing a hydrophobic patch that leads to polymerization in individuals homozygous for the mutation. This reduces the elasticity of red blood cells, causing extreme pain, extensive tissue destruction and anemia. The allele is maintained at a high level in large swaths of African populations because, in the heterozygous state, it affords some protection against the malaria parasite, which replicates in red blood cells (Aidoo et al., 2002). In this case, a single mutation leads directly to a disease that is well understood (albeit imperfectly controlled); however, the relationship between genetic changes, protein misfolding and disease is not always so straightforward.

In this short ‘At a Glance’ piece, we illustrate what happens when proteins misfold and defensive homeostasis mechanisms are unable to keep up with the protein-folding burdens, leading to devastating human disease. Protein misfolding is now implicated in the progression of hundreds of diseases; indeed, it is involved in the majority of diseases not caused by an infectious agent. Our aim is to illustrate the breadth and diversity of the problem, using a select number of diseases with diverse underlying mechanisms. The examples we provide include diseases caused by loss-of-function mutations (due to improper folding, degradation or localization) and diseases resulting from gain-of-function mechanisms (mutations that cause a toxic novel function, dominant-negative mutations and amyloid accumulation). There are many other examples of protein-misfolding diseases, with more coming to light every year. Alas, therapeutic intervention remains, for the most part, at an early stage, but hope is on the horizon. As outlined for the examples below, understanding more about how misfolded proteins contribute to disease opens new avenues for drug discovery.

**Figure:**
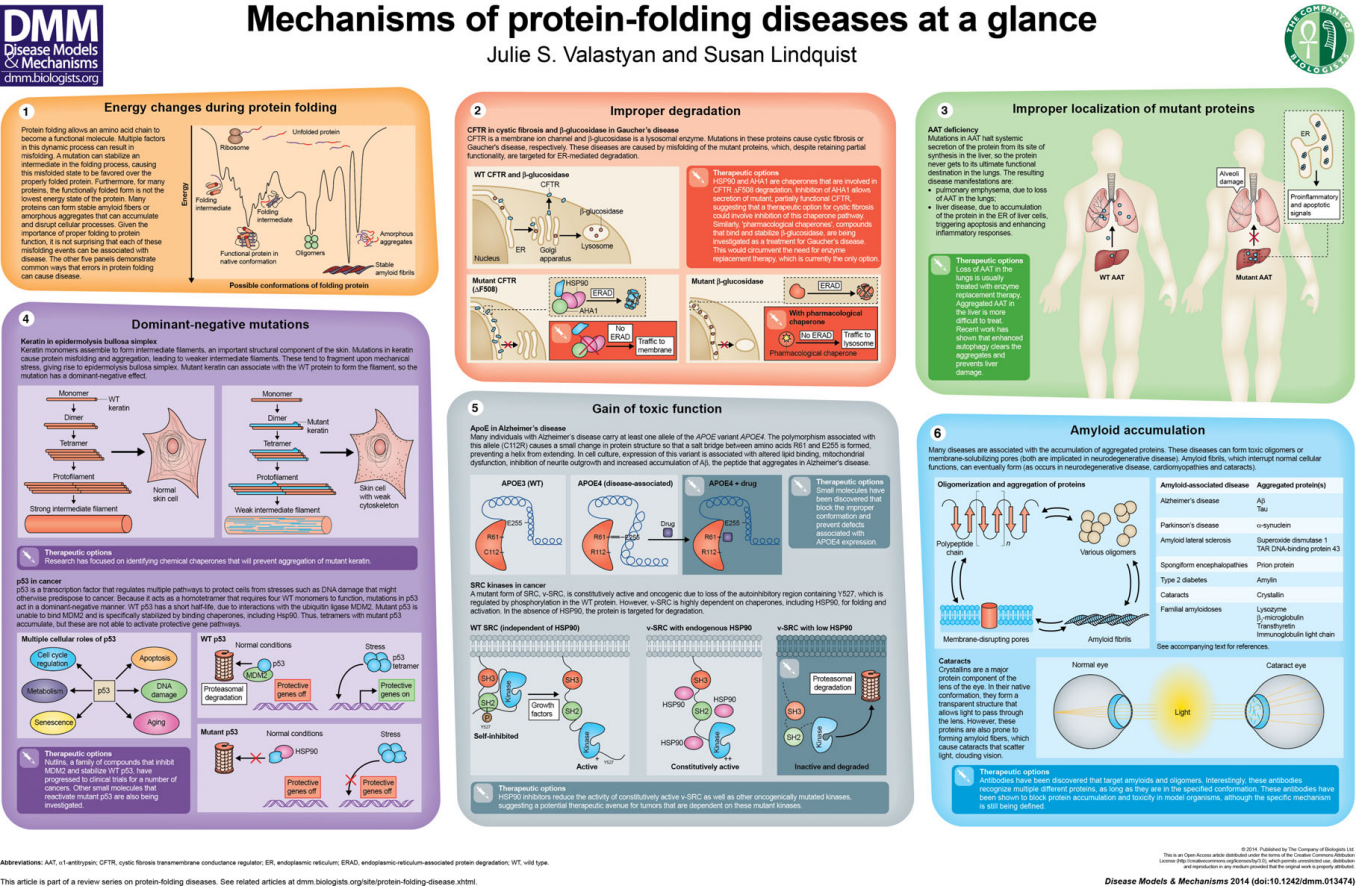


## How protein-folding problems can cause disease

### Improper degradation

Although cellular degradation systems, such as ERAD or autophagy, are essential for preventing the accumulation of non-functional misfolded proteins, they sometimes cause disease by being overactive, degrading proteins that, although mutant, retain some functionality. Thus, improper degradation of proteins can contribute to the development of more severe disease (see poster panel 2). A canonical example is provided by the disease cystic fibrosis, which is caused by mutations in cystic fibrosis transmembrane conductance regulator (CFTR), a plasma membrane chloride channel. The most common causative mutation in cystic fibrosis is deletion of a phenylalanine residue at position 508 (ΔF508) in CFTR. This mutation causes the protein to be misfolded and targeted for degradation (Qu et al., 1997).

The process of CFTR maturation and degradation requires association with multiple chaperones and co-chaperones. Disrupting the function of these chaperone systems can allow mutant CFTR to escape degradation. Upon knockdown of AHA1, a co-chaperone that, together with HSP90, alters the maturation of CFTR, CFTR ΔF508 is not only more stable, but partially functional (Wang et al., 2006). AHA1 is not the only protein that interacts with chaperones to mediate the folding of CFTR. CHIP, a co-chaperone of HSP70, aids in the ubiquitylation and later degradation of mutant CFTR (Meacham et al., 2001); therefore, blocking CHIP function might also allow more CFTR to mature and function. These studies suggest that inhibition of chaperone systems could be therapeutically beneficial to individuals with this mutation.

A related example of this category of protein-folding diseases is provided by Gaucher’s disease, the most common lysosomal storage disease (Futerman and van Meer, 2004; Cox and Cachon-Gonzalez, 2012). Gaucher’s disease is caused by a variety of mutations in β-glucosidase (also known as β-glucocerebrosidase), a lysosomal enzyme with a role in metabolism of the lipid glucosylceramide. Defects in this enzyme result in intracellular accumulation of its substrate, particularly in white blood cells. The symptoms of Gaucher’s disease, which can include bone lesions and enlarged spleen and liver, show a high degree of variability (Grabowski, 2008). Although this variability is not yet fully understood, it is thought to be related to the extent of degradation of β-glucosidase in the ER (Ron and Horowitz, 2005). Depending on the extent of ER processing, some fraction of the protein can be correctly processed, trafficked to the lysosome and retain functionality, even in individuals carrying disease-associated mutations. This has led to the hypothesis that a useful intervention for Gaucher’s disease could be the upregulation of chaperones that assist in the correct folding of β-glucosidase (Sawkar et al., 2006). Indeed, some drugs have demonstrated efficacy in cells derived from individuals with Gaucher’s disease (Sawkar et al., 2002; Sawkar et al., 2005), including drugs that activate the UPR and drugs identified as ‘pharmacological chaperones’, which mimic the activity of a protein chaperone by directly binding a protein. These chaperones function by binding to the enzyme and stabilizing its fold, allowing it to reach its site of activity, i.e. the lysosome. At the lysosome, the pharmacological chaperone is displaced by glucosylceramide, the enzyme’s physiological substrate, which is then successfully processed to its active state. Currently, enzyme replacement is one of the main treatment options for Gaucher’s disease; however, this requires intravenous delivery and is quite expensive. These recent advances suggest that small-molecule-based therapy, which is potentially cheaper and easier to administer than enzyme delivery, could provide an alternative strategy.

### Improper localization

Because many proteins that localize to specific organelles must fold correctly in order to be trafficked properly, mutations that destabilize the correct fold can lead to improper subcellular localization. This can result in dysfunction via both loss of function of the protein at its appropriate location as well as gain-of-function toxicity if it accumulates in an incorrect location (see poster panel 3). One example of this dual toxicity is provided by α1-antitrypsin, a secreted protease inhibitor that, when mutated, leads to emphysema in a recessive loss-of-function manner and liver damage in a dominant gain-of-function manner (reviewed in Perlmutter, 2011). Mutant forms of this protein fail to complete proper folding and are retained in the ER. The misfolded protein is not degraded, unlike other misfolded proteins, so it accumulates in the ER of hepatocytes – the site of synthesis – resulting in liver damage (Lomas et al., 1992; Hidvegi et al., 2005). Furthermore, because the mutated α1-antitrypsin is not secreted, it is unable to perform its normal cellular function, which is to inhibit the action of proteases, including neutrophil elastase, in the lung. This, in turn, causes extensive damage to the lung’s connective tissue. Although damage to the lungs can be controlled with enzyme replacement therapy (Mohanka et al., 2012), liver accumulation has proven a greater medical challenge. However, some progress is being made in light of recent findings. Because aggregates in the liver are degraded by macroautophagy, drugs that enhance autophagy, including rapamycin and carbamazepine, alleviate α1-antitrypsin-induced hepatic toxicity (Hidvegi et al., 2010). Another therapy has focused on directly blocking the aggregation of mutant α1-antitrypsin (Skinner et al., 1998; Mallya et al., 2007).

### Dominant-negative mutations

A third way by which protein misfolding can cause disease is through a dominant-negative mechanism, which occurs when a mutant protein antagonizes the function of the wild-type (WT) protein, causing a loss of protein activity even in a heterozygote (see poster panel 4). In epidermolysis bullosa simplex, an inherited connective tissue disorder, mutant forms of the keratin proteins KRT5 and KRT14 lead to severe blistering of the skin in response to injury. Keratin forms long intermediate filaments that provide structure to the epidermis of the skin (Chamcheu et al., 2011a). Disease-associated mutations in keratin cause the protein to misfold and aggregate, particularly in response to mechanical stress (Russell et al., 2004; Werner et al., 2004). Because a filament is constructed of multiple keratin molecules, a heterozygote individual will make filaments with both WT and mutant versions of the protein. The dominant nature of the disease is therefore explained by the fact that the mutant protein present in these filaments does not function properly, thus compromising the function of the entire filament. As in several other diseases discussed here, recent research has identified chemical chaperones that could prevent the aggregation of mutant keratin and alleviate symptoms of the disease (Chamcheu et al., 2012). The mechanism of action of these compounds is not completely understood. However, one of these compounds, 4-phenylbutyrate (4-PBA), has been found to cause the degradation of aggregated keratin, possibly by increasing the cellular concentration of protein chaperones (Chamcheu et al., 2011b). Treatment with 4-PBA both decreases the total amount of aggregated keratin and increases the amount of HSP70 colocalization with remaining keratin, suggesting that activation of HSP70 by 4-PBA is important for keratin degradation. Crucially, the drug has already been approved for use – it is used to treat other disorders (Maestri et al., 1996) – making it even easier to move it along the pipeline of drug development.

A second example of dominant-negative mutations that involve protein misfolding and predispose individuals to disease is the homotetrameric transcription factor p53. Mutations in p53 are one of the most common genetic alterations seen in cancer (Friedman et al., 1993). Because p53 is responsible for regulating a host of pathways involved in maintaining genome integrity, including apoptosis, DNA damage repair, cell cycle regulation and metabolism (Freed-Pastor and Prives, 2012), mutations in this protein can have far-reaching effects caused by the dominant-negative mechanism outlined below.

Normally, in the absence of genotoxic stresses, p53 is rapidly degraded by the proteasome in a process that is dependent on the ubiquitin ligase MDM2 (Kubbutat et al., 1997). In response to stresses such as DNA damage, p53 is stabilized and then able to stimulate transcription of its target genes. Many of the most common oncogenic mutations in p53 disrupt the core domain of the protein, preventing it from assuming its correctly folded conformation. These mutations have two effects, the overall consequence of which is lack of expression of genes that protect the genome against damage, increasing the risk of cancer. First, although the mutant p53 is still able to associate with other p53 monomers, the resulting tetramer does not function correctly, even if a WT copy of p53 is also present (Milner and Medcalf, 1991; Milner et al., 1991). Thus, mutant p53 acts in a dominant-negative manner; in the heterozygous state, most tetramers are dysfunctional. Second, mutant p53 is unable to interact with MDM2, and thus is not degraded. In addition, mutant p53 is stabilized by binding chaperones, including HSP90. This inappropriate accumulation of the mutant form of the protein makes it even less likely that a tetramer comprised solely of WT p53 will form.

One family of small molecules currently undergoing clinical trial for cancers dependent on p53 dysfunction are Nutlins. These compounds prevent MDM2 from interacting with and promoting degradation of WT p53, increasing the probability of forming WT, functional tetramers (Vassilev et al., 2004). Recently, small molecules that directly bind mutant p53 and restore its function to normal levels have also been found (Gavrin et al., 2012). Given the prominent role of p53 in different cancers, multiple groups have discovered different compounds that restore function of mutant p53. The mechanism by which most of these compounds work is still under investigation; however, the mechanism of action of one compound, pk7088, is well understood. This compound binds and stabilizes a particular p53 mutant, Y220C, and restores its transcriptional functions to that of the WT protein (Liu et al., 2013). Although Y220C represents only a fraction of mutations in p53 that lead to cancer, this protein is so frequently mutated in cancer that even this fraction represents a large patient population that could be effectively treated with this compound. Furthermore, the fact that an individual with cancer caused by a specific mutation should be treated with a specific compound highlights the important role of personalized medicine in cancer treatment.

### Gain of toxic function

Protein conformational changes can also cause dominant phenotypes by causing a protein to acquire a conformation that contributes to toxicity, as illustrated in poster panel 5. One example is apolipoprotein E (APOE), a lipid transport molecule. At least one copy of the *APOE4* allele is found in 65–80% of individuals with Alzheimer’s disease (AD) (Farrer et al., 1997). The polymorphism in *APOE4* stabilizes an altered conformational fold of the protein; the other alleles of this protein have an extended domain structure that is compromised by an extra salt bridge in APOE4 (Dong et al., 1994; Dong and Weisgraber, 1996). This interaction changes the lipid affinity of APOE4 (Dong et al., 1994; Dong and Weisgraber, 1996), disrupts mitochondrial function (Chen et al., 2011) and impairs neurite outgrowth (Nathan et al., 1994).

The *APOE4* polymorphism is also associated with increased levels of Aβ, the peptide that aggregates in the brain of individuals with AD (Ma et al., 1994). Unfortunately, the mechanism for this change is not completely understood, but this association strongly implicates APOE in the pathogenesis of AD. Owing to the specific change in APOE4 structure, small molecules that prevent formation of the extra salt bridge might provide a therapeutic strategy for correcting the dysfunction of this protein. A recent study utilized a FRET-based assay to identify structure correctors that prevented APOE4 from forming the aberrant salt bridge that stabilizes its misfolded form (Brodbeck et al., 2011). Compounds that corrected APOE4 misfolding also rescued APOE4-associated mitochondrial dysfunction and relieved inhibition of neurite outgrowth.

A very different group of proteins that acquire novel pathological functions through mutation are the many oncogenic proteins that drive a great diversity of cancers. The first of these to be identified affected the gene encoding SRC, non-receptor tyrosine kinase. The mutant v-SRC lacks the protein’s normal self-inhibitory phosphorylation site and promotes cell proliferation in an uncontrolled manner. Although v-SRC is constitutively active, it is also much less stable than c-SRC, the WT protein. The oncogenic mutant takes advantage of the fact that the HSP90 chaperone protein provides a protein-folding ‘reserve’ or ‘buffer’. It helps v-SRC acquire its mature fold, localize to the membrane and avoid degradation. Wild-type SRC is much less HSP90-dependent (Xu and Lindquist, 1993; Whitesell et al., 1994; Xu et al., 1999; Bijlmakers and Marsh, 2000). Thus, it is the excess capacity of the HSP90 folding buffer that potentiates the evolution of v-SRC’s malignant phenotype. [In fact, HSP90 plays a large role in the evolution of new phenotypes in all eukaryotes (Jarosz et al., 2010).] Importantly, many other mutated oncogenic kinases, including other SRC family kinases, BCR-ABL (a fusion protein associated with chronic myelogenous leukemia) and BRAF (a serine/threonine kinase that is frequently mutated in melanomas), display this same general problem in protein folding and require similar assistance from the HSP90-based chaperone machinery to exert their malignant phenotypes (for a recent review, see Trepel et al., 2010). The differential requirements that the mutated oncogenic kinases display for HSP90, compared with their normal cellular counterparts, have led to extensive efforts to understand HSP90 function in the treatment of cancers (Whitesell et al., 2012; Taipale et al., 2013). HSP90 is only one of the protein homeostatic mechanisms that contribute to cancer. Recent work highlights the many different ways that cancers subvert the ancient pro-growth and survival functions of the HSR (regulated by HSF1) to promote the malignant phenotype, to the detriment of the host.

### Amyloid accumulation

No review of misfolded proteins and disease would be complete without a discussion of the ability of stable amyloid fibers – insoluble fibrous protein aggregates – to accumulate and contribute to a variety of diseases (see poster panel 6). A range of so-called ‘amyloidogenic’ proteins can cause amyloid-related diseases, and such diseases are classified based on the presence of similar toxic protein conformations. The formation of these protein conformations can lead to a variety of very different diseases (see table in poster panel 6). These range from neurodegenerative disorders (including AD, Parkinson’s disease and Huntington’s disease) to amyloidoses (such as familial amyloid polyneuropathy and primary systemic amyloidosis) (Caughey and Lansbury, 2003; Chiti and Dobson, 2006). In some cases, disease is caused directly by fibril accumulation: a prime example being cataracts (Surguchev and Surguchov, 2010). In other cases, notably the neurodegenerative diseases, the actual cause is much less clear. Lower-order oligomers are now frequently posited as being responsible for disrupting cellular functions. In this scenario, the amyloid deposits could be a protective mechanism that the cell uses to sequester these toxic species (Treusch et al., 2009; Wolfe and Cyr, 2011). However, the amyloid itself could also play a role in spreading the disease from neuron to neuron, perhaps causing yet more havoc (Luk et al., 2012; Nath et al., 2012; Iba et al., 2013). Several of these proteins are also capable of forming pore-like structures that are hypothesized to disrupt membrane integrity, another potential mechanism of toxicity (Lashuel et al., 2002). As previously discussed for α1-antitrypsin deficiency, when pathogenesis involves the accumulation of protein aggregates, it is possible to limit disease by blocking aggregate formation. Because amyloid and other pre-amyloid conformers accumulate in such a wide range of diseases and share structural features with fibril formation, a great deal of work has focused on creating therapeutics that target amyloid folds in general, as opposed to targeting specific proteins. Indeed, antibodies have recently been developed that generically recognize both amyloid fibrils and toxic oligomers, and it has been shown that an oligomer-specific antibody blocks the toxicity of multiple types of oligomers *in vitro* (Kayed et al., 2003; Glabe, 2004). Recent work has turned to developing antibodies that recognize both conformation and sequence, possibly allowing for more specifically targeted therapeutics (Perchiacca et al., 2012). Small molecules that prevent aggregate formation (Ehrnhoefer et al., 2008) or enhance their degradation (Rubinsztein et al., 2007) have also been discovered.

Transthyretin (TTR) provides a particularly impressive example of how knowledge of protein-misfolding mechanisms can provide insights into drug development and lead to treatments for diseases involving amyloidogenic proteins. TTR, which is the primary carrier of the hormone thyroxine and a transporter of retinol, is normally a secreted protein. However, it is amyloidogenic and can form fibrils that can accumulate throughout the body, including the nervous system and heart. The fibrils directly disrupt organ function; however, other pre-amyloid misfolded forms of the protein are also implicated in disease pathophysiology (Gavrin et al., 2012). Misfolded forms of the protein are frequently observed in the elderly, but such forms arise much earlier in life in individuals with mutations in the protein. Early-onset familial cases are associated with devastating amyloidosis diseases such as transthyretin familial amyloid polyneuropathy (TTR-FAP), which is characterized by pain, muscular weakness and autonomic dysfunction.

Insight into how mutant TTR causes disease can be found by looking at the quaternary structure of the protein. The active form of the protein is a tetramer; however, point mutations destabilize the tetrameric form, leading to the accumulation of the monomeric form, which seeds amyloid formation. However, not everyone with the mutant form of the protein develops the disease. A key insight was revealed by the analysis of naturally occurring TTR polymorphisms in healthy individuals. These variants form mixed tetramers with the mutant proteins and prevent disease in individuals carrying these devastating mutations. This led to strategies for stabilizing the tetramer by other means, specifically by binding ligands to the site of its natural thyroxine-binding site. The small molecules that stabilize the tetramer have been approved as a therapeutic intervention in Europe (subject to FDA approval). This is a great success story resulting from the application of deep knowledge of protein-folding problems to the alleviation of a deadly disease (Razavi et al., 2003; Sekijima et al., 2006; Gavrin et al., 2012).

## Tackling the protein-folding problem: conclusions

Herein we have discussed a variety of ways that errors in protein folding lead to disease. Fortunately, current research is unveiling promising avenues to overcome these protein-misfolding events and thereby ameliorate these diseases. Some of these treatment options are protein-specific, whereas many of the therapies involve a more general modulation of chaperones and degradation systems. Because these two systems are integral to cellular protein folding in both pathological and non-pathological states, a better understanding of how they operate as an integrated network is essential to maximize the beneficial effects of therapeutic interventions while minimizing negative side effects. We hope that, as more is understood about the interface between protein misfolding and disease pathogenesis, more specific and innovative treatment options will become available.
